# Acute mastoiditis–associated cerebral sinus venous thrombosis in children: a retrospective review

**DOI:** 10.1007/s00431-025-06477-y

**Published:** 2025-10-02

**Authors:** Yael Sellam, Elchanan Zloczower, Maru Gete, Shoshana Revel-Vilk, Avigail Eisenberg-Wygoda, Gil Lahav, Noam Bartov, Yonatan Lahav, Alex Guri, Ifat Kvint, Eli Shapiro, Amit Nahum, Tal Ben-Ami

**Affiliations:** 1 Department of Pediatrics, Kaplan Medical Center, Rehovot, Israel; 2 Pediatric Hematology-Oncology Unit, Kaplan Medical Center, Rehovot, Israel; 3 Faculty of Medicine, Hebrew University of Jerusalem, Jerusalem, Israel; 4 Department of Otolaryngology-Head and Neck Surgery, Kaplan Medical Center, Rehovot, Israel; 5 Department of Otolaryngology-Head and Neck Surgery, The Eisenberg R&D Authority, Shaare Zedek Medical Center, Jerusalem, Israel; 6 Department of Head and Neck Surgery and Communication Sciences, Duke University, Durham, NC USA; 7 Pediatric Hematology/Oncology Unit, The Eisenberg R&D Authority, Shaare Zedek Medical Center, Jerusalem, Israel; 8 Infectious Disease Unit, Kaplan Medical Center, Rehovot, Israel; 9 Pediatric Neurology Clinic, Kaplan Medical Center, Rehovot, Israel; 10 Pediatric Critical Care Unit, Kaplan Medical Center, Rehovot, Israel

**Keywords:** Cerebral sinus venous thrombosis, Mastoiditis, Anticoagulation, Thrombophilia, Pediatrics, Predictors

## Abstract

Cerebral sinus venous thrombosis (CSVT) is a rare but serious complication of acute mastoiditis in children. Early diagnosis is often delayed due to nonspecific symptoms. This study aimed to identify clinical and laboratory predictors of CSVT in children with mastoiditis to support timely imaging and treatment. We performed a retrospective case–control study across two university-affiliated centers from 1999 to 2022 of children with acute mastoiditis who underwent neuroimaging. Clinical, laboratory, imaging, and treatment data from 81 children (40 with CSVT and 41 without) were compared. Logistic regression was used to identify predictors of CSVT. Four variables were independently associated with CSVT: abnormal neurologic findings (OR 18.24, 95% CI 3.12–150.62, *p* < 0.01), persistent fever > 72 h despite antibiotic treatment (OR 4.67, 95% CI 1.23–19.92, *p* = 0.03), prior antibiotic use (OR 4.74, 95% CI 1.27–21.16, *p* = 0.03), and elevated C-reactive protein (CRP) levels (OR 1.14 per mg/dL, 95% CI 1.05–1.27, *p* = 0.03). The model had strong discrimination (AUC = 0.85). Thrombus resolution was documented in 25 of 32 children (median, 3.8 months). Anticoagulation was safe and effective. Thrombophilia testing had limited diagnostic yield. *Conclusion*: Abnormal neurologic signs, prolonged fever, elevated CRP, and recent antibiotic use are key predictors of CSVT in pediatric mastoiditis. Early imaging and timely anticoagulation contribute to favorable outcomes. Routine thrombophilia screening may have limited utility. These findings may help general pediatricians identify children who need timely referral for neuroimaging and specialist care.

**Table:** 

What is known:
• Cerebral sinus venous thrombosis (CSVT) is a rare complication of mastoiditis in children.
• Diagnosis of CSVT may be delayed due to nonspecific symptoms.
• Anticoagulation is generally safe in children.
What is new:
• We identified four independent clinical predictors of CSVT.
• Early MRI follow-up may allow shorter anticoagulation duration.

## Introduction

Cerebral sinus vein thrombosis (CSVT) is a rare and potentially life-threatening disorder in children that acutely impairs cerebral venous outflow. It can lead to severe complications, including long-term neurological sequelae, and requires prompt diagnosis and treatment. The estimated incidence rate of pediatric CSVT is 0.34–0.67 cases per 100,000 children per year and is higher in neonates compared to children [1–5]. CSVT in children outside the neonatal period presents with a wide spectrum of symptoms, including toxic appearance, headache, seizures, paresis, papilledema, and altered mental status [6–9]. Most children diagnosed with CSVT have favorable outcomes, although chronic disability is reported in 25–74% of children with CSVT, and mortality rates range from 4 to 12% [2, 6, 9–12]. However, there is considerable heterogeneity among study populations, etiologies of CSVT, and outcome reports. Additionally, the lack of standardized outcome measurements and the generally short follow-up durations in most studies further complicate the interpretation of these findings.

Most cases of CSVT in otherwise healthy children are associated with acute head and neck infections, particularly as a complication of acute otitis media (AOM) by mastoiditis [2–4, 9]. While the majority of AOM cases are mild and self-limiting, intracranial complications such as meningitis, epidural or intracerebral abscesses, mastoiditis, and, rarely, CSVT may occur in approximately 0.3–3% of cases. However, reported rates of otogenic CSVT vary depending on the population studied and the definitions of complications used [1, 13–15]. Neurological signs of otogenic CSVT in children are often nonspecific, and in very young children, mild neurological symptoms can be misattributed to concurrent acute infections [8, 16–18]. Imaging studies are crucial for diagnosing CSVT, with magnetic resonance imaging (MRI) being the preferred modality [19]. However, MRI is not routinely performed in acute settings and is not universally available. Consequently, CSVT is most frequently diagnosed using contrast-enhanced computed tomography (CT) scans [16, 20–22].

The treatment for otogenic CSVT includes antibiotic therapy, along with either conservative or more extensive surgical interventions. Although the use of anticoagulation in pediatric otogenic CSVT was previously debated, and despite the absence of randomized control trials in children, major guidelines in both the USA and Europe now recommend its use. These recommendations are based on observational studies, expert opinion, and extrapolation from data in adult populations [19, 20, 23–28]. Anticoagulation agents in the acute phase include unfractionated heparin (UFH), or low-molecular-weight heparin (LMWH), followed by continued treatment with LMWH or vitamin K antagonists. Recent studies have also evaluated the use of oral anticoagulants (DOACs) in the management of pediatric CSVT. Both the direct thrombin inhibitor dabigatran and the direct factor Xa inhibitor rivaroxaban are now approved for the treatment of pediatric venous thromboembolism (VTE), based on their equivalence in efficacy and safety compared to standard of care [24, 29, 30]. However, the optimal duration of anticoagulation remains unclear, with current data supporting a treatment duration ranging from 6 weeks to 6 months [20, 25, 31].

In this study, we aimed to evaluate differences in clinical presentation, laboratory markers, and infectious agents between children with acute mastoiditis, with or without associated CSVT. Our primary objective was to identify risk factors for mastoiditis-related CSVT in children in order to improve the diagnosis and early treatment of this rare complication.

## Methods

This retrospective, two-center case–control study was conducted at two university-affiliated medical centers. The study population included children (< 18 years) diagnosed with acute mastoiditis between 1999 and 2022 who underwent neuroimaging (CT or MRI) during their initial hospitalization. Children without neuroimaging were excluded because CSVT status could not be ascertained reliably. Indications for neuroimaging were categorized into three groups: (1) persistent fever, defined as a fever lasting more than 72 h after starting parenteral antibiotic therapy and/or a surgical procedure; (2) abnormal neurologic findings, either at presentation or during hospitalization, identified by the treating pediatrician and including specific abnormal neurologic findings (e.g., cranial nerve deficits, ataxia, meningismus) or a global assessment of altered mental status or lethargy; and (3) evaluation by the otorhinolaryngology team for cases with clinical deterioration or lack of improvement at the infection site despite appropriate antibiotic treatment, raising concern for local extension of infection (e.g., subperiosteal abscess, petrositis, or intracranial spread).

### Study population

**Fig. 1:**
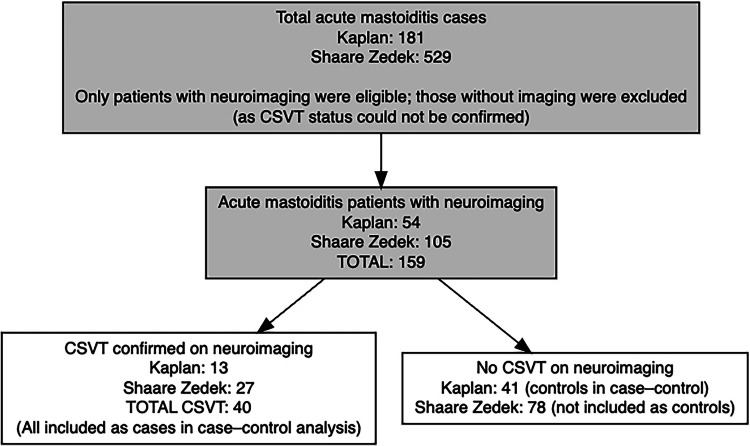
Study population flowchart

During the study period, a total of 181 children at Kaplan Medical Center and 529 at Shaare Zedek Medical Center were hospitalized with acute mastoiditis. Among these, only those who underwent neuroimaging (*n* = 54 at Kaplan, *n* = 105 at Shaare Zedek) were included in the study cohort. Children without neuroimaging were not analyzed, and therefore, the proportion of CSVT among imaged children is not an estimate of incidence among all mastoiditis admissions. For the case–control analysis, we compared children with mastoiditis-related CSVT confirmed on neuroimaging (cases) to children with mastoiditis who underwent neuroimaging but had no evidence of CSVT (controls). All children, from both centers, diagnosed with CSVT (*n* = 40) were included as cases. Children from Kaplan Medical Center (*n* = 41) who underwent neuroimaging during the same period and were not diagnosed with CSVT were included as controls, without additional selection (Fig. 1). No differences in the management of acute mastoiditis were noted between the two centers.

Data were extracted from electronic medical records and included dates of AOM and mastoiditis diagnoses, presenting signs and symptoms, inflammatory markers, radiologic findings (CT and MRI), antibiotic regimens, surgical interventions, neurologic complications, hearing test results, thrombophilia workups, microbiological cultures, viral studies, vaccination history, and clinical outcomes. The institutional review boards of both medical centers approved the study design.

### Statistical methods

Continuous variables were summarized using means and standard deviations or median and ranges, while categorical variables were summarized as counts and percentages. Between-group comparisons were conducted using Student’s *t*-tests and Mann–Whitney *U* test, as applicable for continuous variables, and chi-square or Fisher’s exact tests for categorical variables, as appropriate. Univariate and multivariable logistic regression models were used to quantify the effect of potential risk factors on the probability of CSVT. Variables associated with CSVT at a significance level of *p* < 0.1 in univariate analysis were selected for multivariate modeling. Statistical significance was set at a two-sided *p*-value of < 0.05. All statistical analyses were conducted using the R software (version 2024.12. R Foundation for Statistical Computing, Vienna, Austria).

## Results

The study included 81 children (34 boys), with a median age of approximately 2 years, with a wide age range (Table 1). There were no significant differences in age or sex between children with or without CSVT (Table 1). Similarly, no differences were observed between the groups in terms of underlying rates of recurrent otitis media, prior vaccination status, or antibiotic treatment before hospital admission. The time from admission to CT imaging was comparable between groups, with a median of 3 days. However, the indication for imaging differed significantly. Most children in the control group underwent CT based on expert otorhinolaryngology evaluation, whereas children diagnosed with CSVT were imaged mainly due to persistent fever (Table 1). The majority of children with mastoiditis had a normal neurological examination at presentation, slightly higher in those without CSVT (Table 1).

**Table 1:** Comparison of demographic, clinical, and laboratory characteristics between children with and without CSVT

	Overall *N* = 81	CSVT *N* = 40	Control *N* = 41	*p*-value
Males, *n* (%)	36 (44.4)	16 (40)	20 (48.8)	0.568
Age, months*	22 (4–156)	20 (4–156)	22 (8–130)	0.657
Recurrent ear infections, *n* (%)	23 (28.4)	13 (32.5)	10 (24.4)	0.574
Fully vaccinated, *n* (%)	58 (71.6)	26 (65)	32 (78)	0.399
Antibiotic treatment prior to admission, *n* (%)	49 (60.5)	28 (70)	21 (51.2)	0.133
Abnormal neurologic findings, *n* (%)	15 (18.5)	11 (27.5)	4 (9.8)	0.077
CRP, mg/dL*	15 (0.95–48.4)	17.81 (2.9–48.4)	11.25 (0.95–31.8)	0.004
WBC × 10⁹/L*	17.5 (2.4–36)	16 (2.4–36)	18.4 (5.8–31.4)	0.272
Days for admission to CT*	3.0 (0–17)	3 (0–17)	3.0 (1–8)	0.152
Indication for CT				0.002
a. Prolonged fever	29 (36.7)	20 (50)	9 (23.1)	
b. Neurological symptoms	15 (19)	10 (25)	5 (12.8)	
c. Otorhinolaryngology evaluation	35 (44.3)	10 (25)	25 (64.1)	
Follow-up duration, months*	28.5 (0–192)	24 (1–144)	44 (0–192)	0.218

*CSVT* cerebral sinus venous thrombosis, *CRP* C reactive orotein, *WBC* white blood cells, *CT* computed tomography

^*^Median (range)

**Fig. 2:**
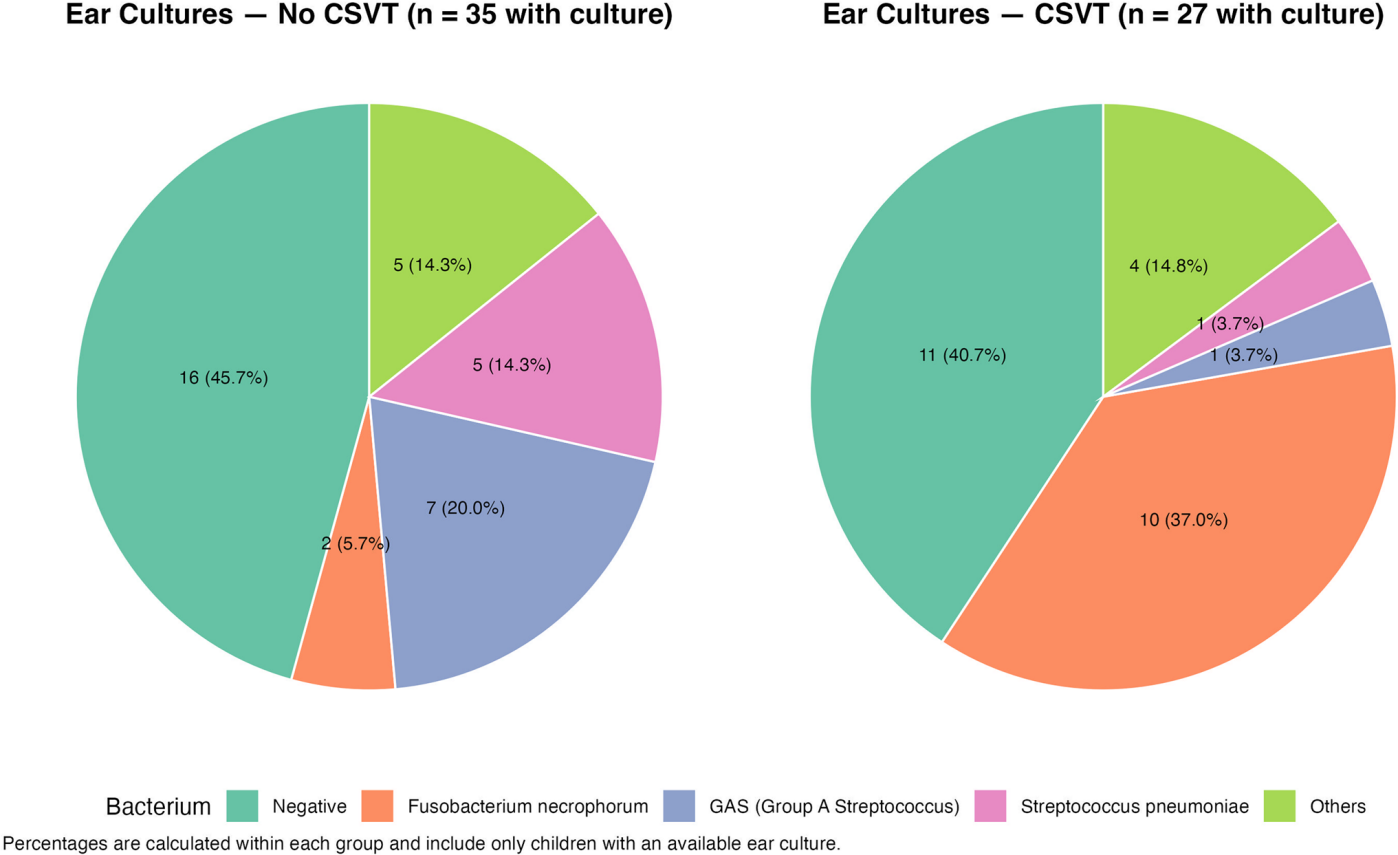
Distribution of bacteria isolated from ear cultures among children with mastoiditis-associated CSVT and those without CSVT. Each pie chart illustrates the relative frequency and percentage of bacterial isolates. “Others” includes organisms isolated in fewer than five patients across both groups

Bacterial cultures, obtained either from middle ear aspirates or mastoid drainage, were available for 62 children. Of these, 35 yielded positive results, with no significant difference in positive culture between the CSVT and non-CSVT groups. However, the type of bacteria found in aspirates differed between the groups. *Fusobacterium necrophorum* was significantly more frequent in the CSVT group (37.0% vs. 5.7%), while *group A streptococcus* (GAS) was more common in the non-CSVT group (20% vs. 3.7%), *p* < 0.001 (Fig. 2). Notably, 43.5% of all cultures were negative, likely due to prior initiation of parenteral antibiotic therapy, potentially reducing culture yield.

All children with mastoiditis-related CSVT received parenteral antibiotic treatment and underwent mastoidectomy as part of their management. Data on anticoagulation were available for 26 children of whom 25 were treated with LMWH. One additional child was documented as not having received anticoagulation; however, no explanation for this deviation was recorded in the medical chart. The median (range) duration of anticoagulation was 3.8 (1.5–12) months. Follow-up MRI was performed in 32 patients at a median (range) of 3.8 (1–9) months, with documented thrombus resolution in 25 cases. None of the children had major bleeding at diagnosis or follow-up. Laboratory evaluation for genetic and acquired thrombophilia was performed in 33 children; 11 had evidence for transient protein S deficiency (*n* = 5), protein C deficiency (*n* = 3), or positive lupus anticoagulants (*n* = 3). Three children with positive lupus anticoagulants and one boy with protein S deficiency were not retested at follow-up. Two children were found to be heterozygotes for factor V Leiden (FVL). On follow-up, two children with CSVT and five children without CSVT had abnormal hearing examinations. One child with CSVT had sixth cranial nerve palsy. There were no clinically meaningful differences in demographic or clinical characteristics between the two participating hospitals. Both institutions followed similar imaging practices to confirm CSVT, and adhered to comparable treatments for mastoiditis and CSVT, supporting the validity of pooling data from both sites.

Risk factor analysis for CSVT was performed using both univariate and multivariate approaches (Table 2). Univariate logistic regression analysis identified four clinical variables associated with CSVT in children with mastoiditis: abnormal neurologic findings, prolonged fever, prior antibiotic treatment, and elevated CRP levels with no evidence for multicollinearity. These four variables were subsequently included in a multivariable model, where all four remained independently associated with CSVT. Abnormal neurologic findings emerged as the strongest predictor. White blood cell count was not significantly associated with CSVT and was excluded from the final model. The final model performed well, demonstrating excellent discrimination (AUC 0.85) and good overall fit (AIC 71.4),

**Table 2:** Univariate and multivariate logistic regression for predictors of CSVT

	Univariate OR (95% CI)	Univariate p	Multivariate OR (95% CI)	Multivariate *p*
Abnormal neurologic findings	5.00 (1.42–19.77)	0.01	18.24 (3.12–150.62)	< 0.01
Prolonged fever	5.56 (1.96–17.05)	0.002	4.67 (1.23–19.92)	0.03
CRP	1.09 (1.03–1.18)	0.004	1.04 (1.01–1.17)	0.03
Previous antibiotic treatment	2.22 (0.91–5.65)	0.09	4.74 (1.27–21.16)	0.03
WBC count	0.92 (0.9–1.04)	0.42		

*OR* odds ratio, *CI* confidence interval, *CRP* C reactive protein, *WBC* white blood cells

## Discussion

In this retrospective case–control study, we identified clinical and microbiological factors associated with CSVT in children with acute mastoiditis. Abnormal neurologic findings, prolonged fever, elevated CRP levels, prior antibiotic treatment, and the isolation of *Fusobacterium necrophorum* from middle ear aspirates or mastoid drainage were significantly associated with CSVT and may serve as important indicators prompting early neuroimaging in this population. The robust association with abnormal neurologic findings (adjusted OR > 18) highlights the importance of close neurologic monitoring, even in the absence of alarming local findings. While abnormal neurologic findings emerged as the strongest independent predictor of CSVT in our cohort, the wide confidence interval (OR 18.2, 95% CI 3.12–150.62) reflects variability that likely stems from the relatively small number of cases and the low prevalence of neurologic signs at presentation. Although this limits the precision of the effect size estimate, the consistent direction and magnitude of association across univariate and multivariate analyses reinforce the clinical relevance of this finding. Future studies with larger sample sizes are needed to confirm the strength of this association and refine its predictive value. In our cohort, the most common neurologic abnormalities were subtle, primarily lethargy symptoms that are easily overlooked in young children. This observation aligns with the findings of Coutinho et al., who reported that children with CSVT most commonly presented with mild neurologic symptoms such as lethargy [8]. Notably, none of the children in our study presented with seizures, diplopia, or focal neurologic signs on admission, as was previously reported in children with mastoiditis-associated CSVT [16, 32, 33]. However, papilledema and cranial nerve palsy were noted during hospitalization in four children who had normal neurologic evaluation at presentation. As most of the children in our study were very young, we could not effectively evaluate for headaches that were reported in older children, emphasizing the relatively subtle neurologic deficits in younger children [17, 32–34]. Prolonged fever (> 72 h despite parenteral antibiotics) and elevated CRP levels were also significant predictors of CSVT in our analysis, aligning with prior studies demonstrating its association with delayed resolution of infections [16, 35].

Microbiologic findings in our study reveal a significant proportion of negative cultures, likely influenced by the frequent initiation of parenteral antibiotics before sample collection. This observation aligns with prior reports and reinforces the importance of timely sample acquisition [8]. *Fusobacterium necrophorum* was notably more common among CSVT cases, in agreement with previous studies identifying this organism as a virulent pathogen associated with invasive head and neck infections and thrombotic complications [16]. While our study did not demonstrate a statistically significant association between *Fusobacterium necrophorum* and CSVT, its known link to thrombogenic infections, as well as other intracranial complications, supports the practice of empirical antibiotic coverage for anaerobic bacteria in suspected cases. Moreover, isolation of *Fusobacterium necrophorum*, even without definitive causality, should heighten clinical suspicion for CSVT and may aid in decisions regarding early neuroimaging.

The safety and efficacy of anticoagulation therapy were reaffirmed in our study, with none of the children experiencing major bleeding complications during treatment. This aligns with findings from the systematic review by Wong et al., which reported only eight bleeding events among 190 children with otogenic CSVT [36]. This supports international guidelines and prior reports that also demonstrated the safety of LMWH in pediatric CSVT management [16, 17, 19, 26].

Thrombophilia testing yielded variable results, and its clinical utility remains a topic of debate. Only two children in this cohort were identified with genetic thrombophilia, a stark contrast to prior studies reporting higher rates of 20–96% of prothrombotic abnormalities in pediatric CSVT [16, 17, 36, 37]. Acute transient findings, including lupus anticoagulants or protein S deficiency, were more common in our cohort. Variations in the timing and specific laboratory tests included in the thrombophilia evaluation between different studies limit the ability to draw conclusions on the routine evaluation of thrombophilia in mastoiditis-associated CSVT. However, current guidelines increasingly discourage thrombophilia testing in provoked venous thromboembolism, as supported by recent systematic reviews and international consensus statements [26, 38, 39].

Outcomes in our cohort were excellent, with recanalization observed in most of children undergoing follow-up MRI, achieved within a median time of 3 months. High rates of recanalization following mastoiditis-associated CSVT were already reported, although mostly requiring longer treatment duration [34, 36]. The combination of early diagnosis, prompt initiation of anticoagulation therapy, and multidisciplinary management likely contributed to these favorable outcomes. The relatively short median time to recanalization observed in our study highlights the importance of early follow-up imaging after discharge. Prompt imaging at 6 weeks post-diagnosis could identify children who have achieved complete thrombus resolution, potentially allowing for shorter durations of anticoagulation therapy without compromising safety or efficacy. This approach aligns with the findings of the Kids-DOTT trial, which demonstrated noninferiority of a 6-week anticoagulation course compared to 3 months for children with provoked venous thromboembolism, with no significant increase in recurrence or bleeding risks [31].

The strengths of this study include the relatively large sample size for such a rare complication, and the careful selection of controls to match disease severity and surgical intervention. However, our study has several limitations. As a retrospective analysis, it is subject to inherent biases, including incomplete data and variability in medical record documentation. The inclusion of only children with acute mastoiditis who underwent neuroimaging based on physician-determined clinical indications likely selected for more severe cases, which may limit the applicability of our findings to all children with mastoiditis. Moreover, cases were drawn from the centers, whereas controls came from a single center, potentially introducing unmeasured institutional differences. Although the multivariate model showed strong discriminatory ability (AUC = 0.85), the relatively small number of CSVT cases (*n* = 40) raises concerns about overfitting. While VIFs were low and no collinearity was detected, the wide confidence intervals, particularly for neurologic symptoms, suggest limited statistical power. Validation in larger, prospective cohorts is needed to confirm these findings. Finally, despite matching for disease severity, unmeasured confounders may have influenced outcomes, and restricting the study to two centers may limit the generalizability of the results to other settings or populations.

## Conclusion

This study highlights the importance of recognizing subtle neurological abnormalities, prior antibiotic treatment, elevated CRP levels, and prolonged fever as key predictors of CSVT in children with mastoiditis. The final model performed well, suggesting it may serve as a clinically useful tool to support early identification of children at risk for CSVT and guide imaging decisions in complex cases.

Our findings reaffirm the safety and efficacy of anticoagulation therapy, while the limited utility of thrombophilia screening suggests a shift towards more targeted evaluations. Early follow-up imaging at 6 weeks post-diagnosis could enable shorter anticoagulation durations in children with complete thrombus resolution, reducing treatment burden without compromising safety.

## Data Availability

No datasets were generated or analysed during the current study.

## Abbreviations

CSVT: Cerebral sinus venous thrombosis
CT: Computed tomography
MRI: Magnetic resonance imaging
LMWH: Low-molecular-weight heparin
CRP: C reactive protein
WBC: White blood cells
AOM: Acute otitis media
